# Optimising the use of the prostate- specific antigen blood test in asymptomatic men for early prostate cancer detection in primary care: report from a UK clinical consensus

**DOI:** 10.3399/BJGP.2023.0586

**Published:** 2024-07-23

**Authors:** Thomas A Harding, Richard M Martin, Samuel WD Merriel, Robert Jones, Joe M O’Sullivan, Mike Kirby, Oluwabunmi Olajide, Alexander Norman, Jaimin Bhatt, Oliver Hulson, Tanimola Martins, Vincent J Gnanapragasam, Jonathan Aning, Meg Burgess, Derek J Rosario, Nora Pashayan, Abel Tesfai, Natalia Norori, Amy Rylance, Andrew Seggie

**Affiliations:** Population Health Sciences, Bristol Medical School, University of Bristol, Bristol.; Population Health Sciences, Bristol Medical School, University of Bristol, Bristol.; University of Manchester, Manchester.; School of Cancer Sciences, University of Glasgow, Glasgow.; Patrick G Johnston Centre for Cancer Research, School of Medicine, Dentistry and Biomedical Sciences, Queen’s University Belfast, Northern Ireland Cancer Centre, Belfast.; British Society for Sexual Medicine, Bygrave, Hertfordshire.; GP training programme director, Barking, Dagenham & Havering GP Vocational Training Scheme.; Surrey and Sussex Cancer Alliance, Guildford.; Queen Elizabeth University Hospital, Glasgow; honorary clinical senior lecturer, University of Glasgow, Glasgow.; Leeds Teaching Hospitals NHS Trust, Leeds.; University of Exeter Medical School, University of Exeter, Exeter.; University of Cambridge; honorary consultant urologist, Addenbrooke’s Hospital, Cambridge.; Bristol Urological Institute, North Bristol NHS Trust and Population Health Sciences, Bristol Medical School, University of Bristol, Bristol.; Prostate Cancer UK, London.; Sheffield Teaching Hospitals NHS Foundation Trust, Sheffield.; Centre for Cancer Genetic Epidemiology, Department of Public Health and Primary Care, University of Cambridge, Cambridge; honorary professor of applied cancer research, Department of Applied Health Research, Institute of Epidemiology & Health Care, Faculty of Population Health Sciences, University College London, London.; Prostate Cancer UK, London.; Prostate Cancer UK, London.; Prostate Cancer UK, London.; Prostate Cancer UK, London.

**Keywords:** prostate cancer, prostate-specific antigen, screening, primary health care, early detection of cancer, consensus

## Abstract

**Background:**

Screening is not recommended for prostate cancer in the UK. Asymptomatic men aged ≥50 years can request a prostate-specific antigen (PSA) test following counselling on potential harms and benefits. There are areas of clinical uncertainty among GPs, resulting in the content and quality of counselling varying.

**Aim:**

To produce a consensus that can influence guidelines for UK primary care on the optimal use of the PSA test in asymptomatic men for early prostate cancer detection.

**Design and setting:**

Prostate Cancer UK facilitated a RAND/UCLA consensus.

**Method:**

Statements covering five topics were developed with a subgroup of experts. A panel of 15 experts in prostate cancer scored (round one) statements on a scale of one (strongly disagree) to nine (strongly agree). Panellists met to discuss statements before rescoring (round two). A lived experience panel of seven men scored a subset of statements with outcomes fed into the main panel.

**Results:**

Of the initial 94 statements reviewed by the expert panel, a final 48/85 (56%) achieved consensus. In the absence of screening, there was consensus on proactive approaches to initiate discussions about the PSA test with men who were at higher-than-average risk.

**Conclusion:**

Improvements in the prostate cancer diagnostic pathway may have reduced some of the harms associated with PSA testing; however, several areas of uncertainty remain in relation to screening, including optimal PSA thresholds for referral and intervals for retesting. There is consensus on proactive approaches to testing in higher-than-average risk groups. This should prompt a review of current guidelines.

## Introduction

Prostate-specific antigen (PSA) testing for early detection of prostate cancer in asymptomatic men is controversial, remaining a significant challenge for the NHS because of uncertainty that any mortality reduction from PSA-based screening outweighs the impact of overdiagnosis and overtreatment on men’s quality of life and healthcare systems. Prostate cancer is a leading cause of cancer death, causing around 12 000 deaths annually in men in the UK.^1^ PSA testing in asymptomatic men can lead to earlier diagnosis and intervention at a curable stage, before it has metastasised.^2^ Currently, 9000 men have late-stage disease at diagnosis annually in the UK.^1^ Most PSA-detected prostate cancers are localised and indolent, so unlikely to cause harm during a patient’s lifetime.^3^

Screening trials using PSA show mixed mortality results. The European Randomized study of Screening for Prostate Cancer (ERSPC) 16-years follow-up showed a 20% relative risk reduction in prostate cancer-specific mortality (PCSM), across multiple centres with varying screening intensities (for example, 2, 4, and 7 years).^4^ Recently, the UK-based Cluster Randomized Trial of PSA Testing for Prostate Cancer (CAP) showed a small (0.09%) reduction in prostate cancer deaths after a median 15-year follow-up after a single PSA screening invitation.^3^ Both trials showed considerable overdiagnosis and overtreatment, and over-represented White men, making it difficult to draw conclusions about the effectiveness of PSA testing in Black men, whose lifetime risk of developing prostate cancer is double compared with White men.^3^^,^^4^ The ProtecT (Prostate Testing for Cancer and Treatment) trial found that in men with PSA-detected, localised disease, PCSM was approximately 3% at 15 years with no statistically significant difference between randomised treatment groups (active monitoring, radical prostatectomy, and radical radiotherapy). However, a higher incidence of metastatic disease was observed in the active monitoring arm (9.4% compared with 4.7% radical prostatectomy, and 5.0% radical radiotherapy).^5^

**Table:** How this fits in

Current primary care guidelines on prostate-specific antigen (PSA) testing in asymptomatic men do not account for updates in the evidence base, or changes to the prostate cancer diagnostic and treatment pathways. This results in variation and inequity of access to PSA testing across the UK; uncertainties regarding which men are likely to benefit from testing; the frequency of testing; and the role of the digital rectal exam. Furthermore, current guidelines rely on men initiating discussions with their GPs about PSA testing. The consensus from the current study challenges previous PSA testing policy. It recommends proactive approaches in men at higher-than-average risk but does not go as far as recommending population or targeted screening. The authors believe this consensus provides answers to help address some of the inconsistencies of practice and areas of confusion in relation to PSA testing in asymptomatic men, and also highlights additional areas of research needed on this subject. In the absence of up-to-date PSA testing guidelines, the authors hope this consensus can support primary care clinicians in the optimal use of the PSA test in asymptomatic men for the early detection of prostate cancer.

ERSPC, CAP, and ProtecT demonstrate PSA-detected prostate cancers have significant lead times, suggesting screening for prostate cancer may, on average, take 10–15 years or more to deliver meaningful benefits for men.^6^ Long acquisition times for new evidence poses additional challenges for health agencies making recommendations on screening.

In 2020, the UK National Screening Committee upheld its recommendation against population screening with PSA, as evidence did not suggest the benefits outweighed harms.^7^

In the absence of organised screening, the UK operates an informed-choice approach, whereby asymptomatic men aged ≥50 years interested in PSA testing must first be counselled by their GP on the pros and cons. The prostate cancer risk management programme (PCRMP) provides guidance for GPs on how to counsel patients. This process is patient led, with GPs recommended against proactively raising PSA testing with asymptomatic men.^8^ Evidence suggests GPs’ views on PSA are varied, and quality and content of counselling may vary considerably.^9^

Since ERSPC and CAP were published, there have been changes to the prostate cancer diagnostic and treatment pathways. First, the introduction of prebiopsy multiparametric magnetic resonance imaging (mpMRI), which may allow a quarter (27%) of patients to avoid prostate biopsy and reduce diagnosis of low-grade cancers, not requiring radical treatment, by 5%.^11^ In 2018, 86% of NHS trusts and health boards reported offering prebiopsy magnetic resonance imaging (MRI),^12^ before the National Institute for Health and Care Excellence (NICE) recommended it as the standard pathway in 2019. Second, use of mpMRI to target prostate biopsies may improve detection of clinically significant prostate cancers compared with systematic biopsies,^13^ although long-term outcomes data are lacking.^14^ Third, increased uptake of active surveillance in low- and intermediate-risk disease may allow some patients to delay or avoid radical treatment, potentially reducing overtreatment.^15^ These changes have led professional bodies, such as the European Association of Urology, to change their view on PSA-based screening.^16^

However, there is lack of evidence assessing the impact of these changes on long-term outcomes, such as PCSM or metastatic disease incidence reduction, either in opportunistic or organised screening. Furthermore, it has been suggested that MRI-targeted biopsy may increase overtreatment associated with PSA testing by detecting small amounts of Gleason grade 4 cancer that may have been missed by systematic biopsy and are unlikely to cause harm.^17^^–^^19^ This may lead to ‘upgrading’ of patients who would otherwise be considered low risk (Gleason 6 [3 + 3]), changing their management from active surveillance to radical treatment.^19^

Given the ongoing changes within prostate cancer diagnostic and treatment pathways, Prostate Cancer UK commissioned a clinical consensus to ascertain expert opinion on optimal use of the PSA test in asymptomatic patients. The authors of the current study hope this consensus highlights areas of uncertainty, which should be considered priorities for future research and help inform updates to UK policy. Lastly, the aim is to influence changes to guidelines for GPs, other healthcare professionals (HCPs), and asymptomatic men.

## Method

A modified RAND/UCLA appropriateness method^10^ for determining consensus was followed:

- Expert panels were formed — a lived experience (LE) and HCP panel, including researchers and academics.
- Two rapid evidence reviews were conducted:
  1. what are the benefits and harms of PSA-based prostate cancer screening and the diagnostic pathway? — Do benefits and harms vary by risk factors (age/ethnicity/family history)?^20^ And
  2. have benefits and harms of PSA-based prostate cancer screening changed since the introduction of prebiopsy mpMRI and additional testing into the prostate cancer pathway?^14^
- Review of worldwide PSA testing guidelines (see Supplementary Table S1).
- Rapid reviews, guidelines review, and previous PSA consensus were used to draft 94 statements over five topics:
  1. awareness raising, discussions about PSA testing, and informed choice (*n* = 25);
  2. PSA testing and referral for further investigation (*n* = 45);
  3. role of digital rectal examination (DRE) and patient medical history in decision making (*n* = 8);
  4. UK readiness for a targeted prostate cancer screening programme (*n* = 13); and
  5. research opportunities and priorities (*n* = 3).
- Panellists were asked to independently score statements — 1 (strongly disagree) through 5 (neutral) to 9 (strongly agree) using an online survey platform. The LE panel scored a subset of 33 questions from topics 1 and 3.
- Statements alongside median and consensus values were presented back to panels at separate meetings, LE panel first (virtually) then HCP panel (in person). Rapid evidence and international guideline reviews were provided.
- Both panels were able to propose rewording and combining of statements based on discussion and clarification of definitions. Discussion and statement rewording was moderated by the Chair and changes captured by a member of the data and evidence team.
- Insights, including proposed changes to wording and combining of statements from the LE panel, were presented ahead of each topic, to support HCP panel discussions.
- HCP panellists were given opportunity to rescore any statements. Rescoring was based on changes to panellists’ preferences following statement clarification, discussion of evidence, consideration of real-world practice/implementation, and consideration of the LE panel’s preferences.
- Following the HCP panel consensus, values were recalculated using interpercentile range adjusted for symmetry (Box 1).
- Clarification of seven statements was achieved by email.

**Box 1.** Consensus analysis and definition

Following the panels scoring their respective statements, median scores were calculated: 1–3 indicated ‘disagreement’; 4–6 indicated ‘uncertainty’; and 7–9 indicated ‘agreement’.	
Consensus among panellists was calculated by using the interpercentile range adjusted for symmetry (IPRAS).^10^ Lower dispersion of scores among the panel (IPRAS <1) indicates consensus. Upper and lower interpercentile ranges were 15% and 85%, respectively. Consensus between panellists can be reached either in agreement or disagreement with individual statements.	

‘**Consensus**’ was assigned when the following conditions were met: Median 1–3 (disagreement) and IPRAS <1 Median 7–9 (agreement) and IPRAS <1	‘**No consensus**’ was assigned when the following conditions were met: Median 1–3 (disagreement) and IPRAS ≥1 Median 7–9 (agreement) and IPRAS ≥1 Median rating 4–6 (uncertainty) irrespective of IPRAS

## Results

Round one included 94 statements of which 55 (59%) achieved consensus. Following the face-to-face discussion (round two), 48/85 (56%) statements achieved consensus. Individual statements for each topic that did/did not reach consensus varied between rounds. Table 1 shows numbers and percentages of statements reaching consensus, before and after discussion, and summarises statement changes.

**Table 1.** Number of items reaching consensus for each topic, pre- and post-discussion

**Topic**	**Pre-discussion — virtual (round 1)**	**Post-discussion — face to face (round 2)**	**Summary of changes to statements**
	**Consensus, *n*/*N* (%)**	**Consensus, *n*/*N* (%)**	
**1. Awareness raising, discussions about PSA testing, and informed choice**	19/25 (76) 21/25 (84) LE panel	13/15 (87)	21/25 statements rescored by HCP panel during consensus meeting. Statements 1–4, 5–7, and 16–21 combined to create three new statements. Another 6 statements were reworded
**2. PSA testing and referral for further investigation**	21/45 (47)	19/46 (41)	45/45 statements rescored. Ten statements were reworded and 1 new statement was included
**3. Role of the digital rectal examination (DRE) and patient medical history in decision making**	3/8 (38) 4/8 (50) LE panel	4/8 (50)	8/8 statements rescored. Two statements were reworded
**4. UK readiness for a targeted prostate cancer screening programme**	9/13 (69)	9/13 (69)	13/13 statements rescored. No changes^a^
**5. Research opportunities and priorities**	3/3 (100)	3/3 (100)	3/3 statements rescored. No changes^a^
**Total items**	**55/94 (59) 25/33 (76) LE panel**	**48/85 (56)**	

a *‘No changes’ means there were no statements re-worded under topics 4 and 5. All statements presented in the above table were scored by the HCP panel. A subset of 33 statements from topics 1 and 3 were scored by the LE panel. HCP = healthcare professional. LE = lived experience. PSA = prostate-specific antigen.*

Box 2 summarises statements with consensus and agreement.

**Box 2.** Summary of statements achieving consensus and agreement^a^

**Topic 1: Awareness raising, discussions about PSA testing, and informed choice**
***Awareness raising***
•There is a need to raise awareness of prostate cancer among men aged ≥50 years, or from aged 45 years for Black men and men with a family history of prostate cancer
•Responsibility for raising awareness of prostate cancer is shared by charities, the NHS, governments, and public health bodies
***Responsibility for initiating discussions***
Primary care health professionals should proactively discuss prostate cancer risk, PSA testing, and the wider diagnostic pathway with men aged ≥45 years at higher-than-average risk of prostate cancer owing to any of the following: ○ Black ethnicity; ○ a family history of prostate cancer; and ○ confirmed to have genetic risk factors that increase their risk of developing prostate cancer, for example, *BRCA2* gene variations
• Primary care health professionals need to be supported with training and education resources that focus on the factors that put men at higher-than-average risk of prostate cancer, the benefits and harms of the PSA test, and the wider prostate cancer diagnostic and treatment pathways
***Decision making/informed choice***
• Men should be given balanced information on the pros and cons of the PSA blood test and supported to make an informed choice whether to have one. *(That information can come from trained health professionals, charities, or can be delivered via validated online tools to support informed choice)*
• A man’s PSA level should be reviewed alongside other known risk factors to aid in the decision as to whether to refer them on the 2-week-wait suspected urological cancer pathway/secondary care decision-making process
• The individual benefits of a PSA blood test will be different for each man. *(This is because some men will have more risk factors for prostate cancer than others or will have pre-existing health conditions. Information and counselling on the PSA blood test should explain this)*
• Men who are considering PSA testing should be able to discuss the potential benefits and harms with a trained health professional and make an informed decision based on their individual circumstances (for example, comorbidities) and preferences
**Topic 2: PSA testing and referral for further investigation**
• All informed men should have the potential to access the PSA blood test from the age of 50 years
Strongly recommend that the PSA blood test be proactively discussed with any of the following: Black men from the age of 45 years; men from the age of 45 years if they have a known family history of prostate cancer, particularly if a first-degree relative has died at a young age of this cancer; and men from the age of 45 years if they have confirmed genetic risk factors that increase their risk of developing prostate cancer, for example, *BRCA2* gene mutations
• Strongly recommend that for Black men with a known family history of prostate, breast, or ovarian cancer — in particular if a first-degree relative has died at a young age of this cancer — the PSA blood test should be proactively discussed from the age of 45 years
• The individual benefits of the PSA test will be different for each man; trained health professionals should consider overall health status, not just age, when making decisions about PSA testing
• PSA testing should, where possible, be performed under optimal conditions. Before testing, trained health professionals should advise men on factors that could temporarily raise/lower PSA levels, for example, vigorous exercise, sex/ejaculation in previous 48 h, use of medications including finasteride, and active urinary infection
• Frequency of repeat PSA testing should be risk-stratified based on PSA value, age, ethnicity, and family history
• There is a role for the use of PSA velocity in addition to total PSA in determining whether to refer men to secondary care
• For all repeat PSA testing scenarios, men should have an opportunity to discuss the potential benefits and harms of the PSA test with a trained health professional, to include the latest information on the pros and cons of the PSA test, wider diagnostic pathway, and the man’s individual circumstances and preferences
• Future PSA testing intervals should be communicated to the primary care team and patient, and, where possible, should be recorded on the GP system as an alert for follow-up
**Topic 3: Role of digital rectal examination (DRE) and patient medical history in decision making**
• GPs should refer asymptomatic men with a PSA below the threshold if the DRE is suspicious
• Men with a PSA level above the referral threshold do not need to have a DRE prior to referral to secondary care
• A man’s relevant medical history should be included when referring to secondary care for further investigation
**Topic 4: UK readiness for a targeted prostate cancer screening programme**
• Since the 2019 National Institute for Health and Care Excellence guidance update, the prostate cancer diagnosis and treatment pathways have changed to make it safer and more accurate
• The PSA blood test is the first step in the prostate cancer diagnostic pathway. It is a cheap, safe, and effective way of identifying men who would benefit from further testing — in the first instance an MRI scan
• There is not a national screening programme for prostate cancer, so men will not get invited to have a test. Men at risk, who have discussed the harms and the benefits of the PSA test, have a right to a PSA test from a trained health professional if they want one
• The balance of benefits and harms is shifting in favour of screening, but evidence gaps remain, and more research is needed
• More research is required to gather evidence from randomised controlled trials, real-world data, and studies to evaluate the feasibility and effectiveness of the implementation of organised screening programmes using PSA with MRI as follow-up
• In the absence of a prostate cancer screening programme in the UK, the NHS should commission services that enable men at risk of prostate cancer to access PSA testing outside of primary care. These services must be high quality, have appropriate clinical oversight, and communicate effectively with the patient, primary care, and secondary care
• Any increase in PSA testing is likely to pose a substantial challenge to primary care resources. The NHS should provide all necessary resources for men to be able to access the PSA test according to guidance within the prostate cancer risk management programme
• The NHS should consider alternative models of accessing PSA testing with appropriate quality and safety checks to ensure men can make an informed choice about PSA testing
**Topic 5: Research opportunities and priorities**
Future research should prioritise discovery and validation of pathways that: ○ find aggressive cancers that are currently being missed; ○ further reduce harms (unnecessary biopsies and detection of clinically insignificant disease) in men who undergo PSA testing through informed choice; and ○ allow for better risk stratification of men who undergo PSA testing through informed choice

a *This box shows a summary of consensus statements that achieved consensus with agreement following both the lived experience and HCP panels. Supplementary Table S2 shows all statements. HCP = healthcare professional. MRI = magnetic resonance imaging. PSA = prostate-specific antigen.*

### Topic 1: Awareness raising, discussions about PSA testing, and informed choice

Panellists agreed in consensus there is a need to raise awareness of prostate cancer and risk factors among at-risk men in the UK, although some raised questions regarding lack of published evidence on the benefits of awareness. There was consensus that awareness messaging should be targeted at men aged ≥50 years, or ≥45 years in men with a family history of prostate cancer or men of Black ethnicity.

The LE panel discussed issues of placing primary responsibility with one organisation. They proposed a combined statement to give organisations shared responsibility, encouraging greater collaboration, and delivery of joined-up and consistent messaging. The HCP panel agreed that responsibility for raising awareness should be shared by charities, the NHS (primary and secondary care), and governments/public health agencies. A clear distinction was made between raising prostate cancer awareness, and instructing men to undergo PSA testing, with emphasis being placed on the former, empowering men to make informed choices on testing.

The LE panel reached consensus on proactive conversations in all men aged ≥50 years. This may reflect commonly expressed views of men who experience a late diagnosis that, had they known more, they might have been diagnosed earlier. However, there was no agreement within the HCP panel whether prostate cancer risk and PSA testing should be proactively discussed with asymptomatic men aged ≥50 years, in the absence of any other prostate cancer risk factors. Contrary to current UK recommendations, both panels supported proactive conversations between primary care HCPs and patients at higher-than-average risk because of Black ethnicity, or a family history of prostate cancer, or confirmed genetic risk factors (for example, *BRCA2* gene mutation). However, concerns were raised about the feasibility of initiating proactive conversations in men with a family history of prostate cancer or *BRCA2* gene mutation, given the poor recording of both in primary care.

In keeping with current guidelines, HCP panellists agreed only men who have been appropriately counselled should have potential access to a PSA test. Counselling should be performed by ‘trained health professionals’ and include details on the wider prostate cancer diagnostic pathway, that is, mpMRI and biopsy. The LE panel reflected on the need for HCPs to have expertise in counselling men on prostate cancer and PSA testing, and suggested looking beyond GPs to do this. The HCP panel agreed and suggested expanding to ‘trained healthcare professionals’. Panels agreed that primary HCPs need to be supported with training and education resources focusing on: factors that increase prostate cancer risk, benefits and harms of PSA testing, and wider prostate cancer diagnostic and treatment pathways. LE panellists highlighted that details about the wider diagnostic pathway could be ‘frightening for men’, and there was a fine balance between supporting men to make informed decisions and ‘shocking and scaring them’.

### Topic 2: PSA testing and referral for further investigation

HCP panellists agreed that well-informed men should have the potential to access PSA testing from the age of 50 years, noting there may be differences between potential and realised access. Panellists were originally asked whether any risk groups should be ‘proactively offered a PSA test’, but they felt it was more appropriate to modify statements to recommend PSA testing be ‘proactively discussed’ with higher-than-average risk men (Black men, men with a family history of prostate cancer, or men with confirmed genetic risk factor, aged ≥45 years). However, concerns were raised regarding potential for harm because of proactive engagement.

Consensus was not reached on appropriate PSA threshold values because of the lack of high-quality evidence to support age-specific PSA thresholds in asymptomatic patients. Questions were raised regarding the rationale behind the current threshold of ≥3.0 ng/mL for asymptomatic patients aged 50–69 years, but panellists could not reach consensus on alternatives. The impact of risk factors, such as ethnicity, on PSA threshold values was also highlighted as requiring further research.

Similarly, there was no consensus on eligibility or frequency of repeat PSA testing in patients who do not meet the threshold for referral, because of insufficient evidence and concerns over the burden any retesting scenarios may place on primary care. Panellists agreed that frequency of repeat testing should be risk-stratified based on PSA value, age, ethnicity, and family history, but noted risk-stratification may be aspirational and not practical, given the lack of validated risk-stratification tools. There was consensus that patients who do undergo repeat PSA testing should have an opportunity to discuss potential harms and benefits again with a trained HCP. There was consensus that PSA tests should be performed under optimal conditions, with primary care HCPs encouraged to instruct men on factors that may affect PSA levels (for example, urinary tract infection or ejaculation). However, achieving optimal conditions should not create barriers to PSA testing.

Although panellists did not reach consensus on baseline test values, risk of metastatic disease or lethal prostate cancer being low for men aged ≤40 years and men with a baseline PSA <1 ng/mL was recognised. Panellists also highlighted that in men with prostate cancer and a PSA of 3–19 ng/mL the risk of dying from prostate cancer, as shown in ProtecT, was <3%.^5^ Consensus was not reached on whether repeat PSA tests are required in men with a PSA above threshold, before referral, to rule out other causes of a raised PSA, such as urinary tract infection.

### Topic 3: Role of digital rectal examination and patient medical history in decision making

LE panellists discussed personal experiences of DRE and questioned the benefits of the test in detecting prostate cancer over PSA and MRI; they referred to cases where men have ‘a positive PSA and MRI, but no detectable cancer on DRE’. They suggested DRE could be ‘offered’ rather than mandated and should be based on an individual man’s risk factors and preferences.

HCP panellists agreed that men with a PSA above the threshold (≥3.0 ng/mL) do not need to undergo DRE before secondary care referral. Panellists were uncertain over the value of offering a DRE to asymptomatic men with a normal PSA (regardless of risk factors such as family history of prostate cancer or Black ethnicity), given the relatively low positive predictive value of DRE and poor concordance between DREs performed in primary versus secondary care.^21^^–^^23^ However, panellists agreed that, if DRE is done, GPs should refer asymptomatic men with PSA below threshold, if the DRE is suspicious, and a man’s relevant medical history should be included in the referral. No consensus could be reached on whether performing DRE before a PSA test would have an impact on the PSA result (that is, an increase in PSA level).

### Topic 4: UK readiness for a targeted prostate cancer screening programme

There was consensus that, since the 2019 updates to the NICE guidelines, prostate cancer diagnosis and treatment pathways have become safer and more accurate.^24^ Panellists agreed the balance of harms and benefits is shifting towards a lower risk of harms associated with prostate cancer diagnosis and treatment, but important evidence gaps remain. There was agreement that more evidence from both randomised controlled trials and real-world studies were needed to evaluate feasibility and effectiveness of organised screening programmes using PSA with MRI as follow-up. Furthermore, panellists noted that, although current standard pathways are acceptable (for prostate cancer diagnosis), screening requires a clear end-to-end pathway. Panellists did not reach consensus on the acceptability of the PSA with MRI as follow-up in a nationwide targeted screening programme for Black men or men with a family history of prostate cancer.

In the absence of formalised screening, there was consensus that men at risk (aged ≥50 years, Black men aged ≥45 years, and men with family history aged ≥45 years) who have discussed PSA test harms and benefits have the right to a PSA test from their GP. However, there was consensus that any increase in PSA testing would likely pose a substantial challenge to primary care resources and therefore the NHS should provide all necessary resources for men to be able to access PSA tests, according to PCRMP guidelines. Panellists supported NHS-commissioned services that enable men at risk of prostate cancer to access PSA testing outside of primary care, although several panellists raised concerns regarding ‘screening by the back door’ associated with such initiatives.

### Topic 5: Research opportunities and priorities

Three priority areas of research were agreed by HCP panellists, which may help address some of the concerns and uncertainties about PSA testing in asymptomatic men. These were the discovery and validation of pathways that:

- find aggressive cancers that are currently being missed;
- further reduce harms associated with PSA testing; and
- strategies allowing for better risk-stratification of men who undergo PSA testing.

**Figure 1. fig1:**
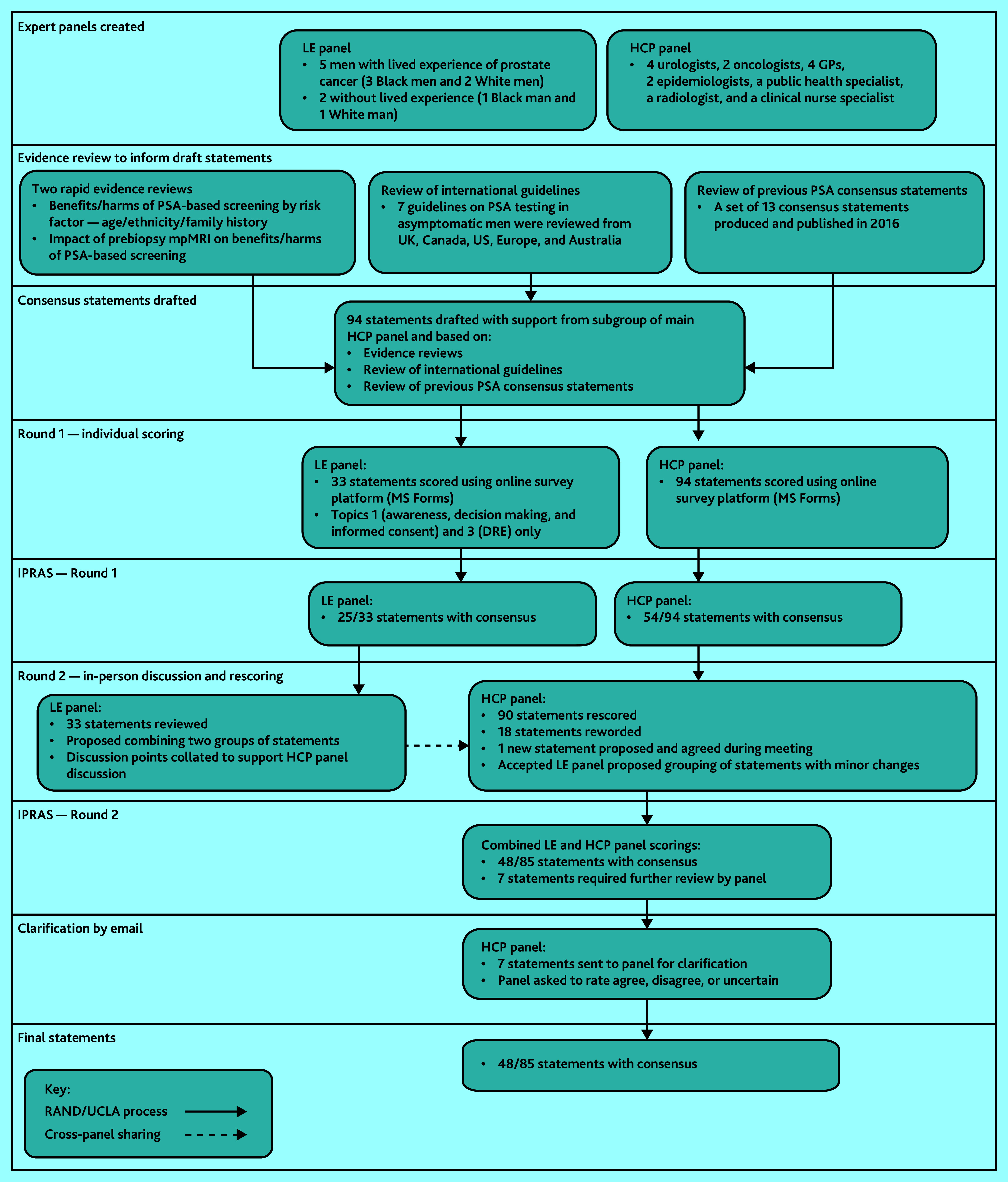
Consensus methodology. DRE = digital rectal examination. HCP = healthcare professional. IPRAS = interpercentile range adjusted for symmetry. LE = lived experience. mpMRI = multiparametric magnetic resonance imaging. PSA = prostate-specific antigen.

Panellists noted the lack of consensus regarding risk-stratification throughout previous topics, highlighting this as a particular requirement of future research. Panellists reiterated that the purpose of any screening should be detection of aggressive cancers carrying high oncological risk, referring once more to ProtecT data, noting that rate of disease progression was low compared with the rate of treatment.^5^[Fig fig1]

## Discussion

### Summary

The current study has presented formal recommendations from a RAND/UCLA consensus outlining the optimal use of the PSA test in asymptomatic men. Recommendations should be considered alongside clinical evidence to guide UK PSA testing policy.

The panel agrees prostate cancer diagnostic pathway improvements have reduced some of the harms associated with PSA testing but several areas of uncertainty remain, such as appropriate PSA referral values, retesting intervals in men who are at higher risk, and the extent to which overdiagnosis or overtreatment have reduced. The panel made several recommendations that conflict with current guidelines, such as proactive rather than reactive conversations about prostate cancer and PSA with men at the highest risk (Black men aged ≥45 years, men with family history of prostate cancer ≥45 years, and men with confirmed genetic risk factors ≥45 years), and recommending against routinely performing DRE in asymptomatic men. These recommendations should be considered by UK health agencies when updating PSA testing policy for patients who are asymptomatic.

### Strengths and limitations

HCP panellists were given two rapid evidence reviews on PSA testing,^13^^,^^20^ and a comparison of current international guidelines on PSA testing (see Supplementary Table S1) to ensure evidence-based discussions throughout. A broad range of relevant expert HCPs and prostate cancer researchers from across the UK were included to encompass a range of viewpoints. A diverse lay panel of men with and without prostate cancer were also included, with significant (*n* = 4/7 [57%]) representation of Black men, which was important as lifetime risk of diagnosis and death in Black men has been reported to be twice that of their White male counterparts.^25^ Insights from LE panellists were presented to the HCP panel during each topic discussion. An independent chair ensured balanced discussions and equal contribution from all participants.

As with any clinical consensus, the current results reflect opinions of the panels, and there is a potential for selection bias. Lay panel members were required to join an online discussion, and this may have excluded patients without adequate IT provision, which may have biased results. Recommendations made by panellists have been made in the context of limited robust evidence and therefore continued focus on the evidence base to support decision making around PSA testing should remain a priority.

### Comparison with existing literature

Current guidelines (PCRMP and NHS) recommend GPs do not proactively raise the issue of PSA testing with asymptomatic men. The consensus from the current study deviates from this guidance, recommending primary care HCPs proactively discuss prostate cancer risk, PSA testing, and the wider diagnostic pathway with men aged ≥45 years at higher-than-average risk (that is, Black ethnicity or family history of prostate cancer, or confirmed genetic risk factors). It did not recommend proactive conversations for all at-risk men — that is, aged ≥50 years without other risk factors. This consensus, in some ways, is reflective of changes to policy in other countries. For example, European Union member states have signed up to a recommendation that countries should consider a stepwise approach on the implementation of organised prostate cancer screening programmes.^26^ More recently, an expert panel in the US recommended that annual screening using the PSA test should be strongly considered for Black men from the age of 40 years.^27^ The review of guidelines undertaken in the current study demonstrates there is still variation in recommendations for men at higher-than-average risk, with most lacking specific recommendations on proactive approaches to testing in Black men and men with a family history of prostate cancer. This should prompt a review of UK guidelines and further agreement on how proactive discussions should be implemented within clinical practice.

### Implications for research and practice

Appropriate PSA referral thresholds for asymptomatic men are an area of uncertainty. Likewise, there is evidence that ethnicity can affect PSA values — with higher values in Black men compared with White men.^28^ Current guidelines do not account for this variation and poor representation of Black men in prostate cancer screening trials meant the current study was unable to reach recommendations regarding threshold values in Black men. Further research exploring sensitivity and specificity of different PSA threshold values for aggressive and/or fatal prostate cancer in Black men is needed. Until new evidence suggests otherwise, the current referral threshold of ≥3.0 ng/mL for asymptomatic men should be used.

Patients with a PSA <3.0 ng/mL may naturally ask about frequency of retesting. HCP panellists were unable to reach specific conclusions, although if patients do undergo retesting they recommended risk-stratified approaches integrating prostate cancer risk factors with previous PSA levels. To the authors’ knowledge, there is no best-practice examples of this and such a recommendation would be reliant on individual GPs’ clinical judgement. There is a need for research into validated stratified follow-up incorporating baseline patient characteristics and initial PSA values.

There was no consensus on the current acceptability of PSA followed by MRI as a screening programme in the UK. Statements on readiness for screening or targeted screening were far from consensus — some strongly agreeing and others strongly disagreeing. Concerns around overdiagnosis and overtreatment associated with PSA testing remain. There is a clear need to develop an effective prostate cancer screening programme to reduce the substantial number of late-stage diagnoses while reducing overdiagnosis of non-aggressive cancers. Clinical trials evaluating PSA testing in the context of diagnostic pathway changes — mpMRI before biopsy and risk-stratified approaches, such as polygenic risk scoring, are needed. Furthermore, recent studies have explored the possibility of using short (biparametric) prostate MRI in combination with PSA density as an initial screening test,^29^ although more research is needed to fully determine the viability of this approach.

Panellists agreed there is a need to raise awareness of prostate cancer and prostate cancer risk factors among at-risk men in the UK, and this responsibility should be shared by charities, the NHS (primary and secondary care), and government/public health agencies. Currently, there is no national or consistent approach to awareness, which risks exacerbating inequalities. There is a need for a coordinated response led by government and public health agencies. PSA testing decisions are complex and the importance of counselling and informed choice was made clear throughout. It is important that ‘trained health professionals’ counselling men on PSA testing are aware of updates to prostate cancer diagnostic and treatment pathways that may reduce harm, while also informing men of the risks of overdiagnosis and overtreatment.

Several panellists expressed concerns that current opportunistic approaches lead to inequality of access to PSA testing, as men must initiate conversations. Indeed, there is evidence demonstrating significant regional variation and lower PSA testing rates in areas of greater deprivation.^30^ Although evidence exists that higher rates of opportunistic PSA testing may reduce diagnosis of metastatic prostate cancer,^31^ whether variation in PSA testing leads to differences in prostate cancer mortality is unclear. The extent to which increasing awareness or proactive conversations may increase overdiagnosis of indolent disease is also unclear.

Implications for broader primary care services must be considered. Counselling patients on PSA testing is complex and time consuming. Some GPs have already expressed concerns that any increases on demand may negatively have an impact on their ability to deliver other aspects of care.^32^ Panellists agreed that, in the absence of screening, the NHS should commission services that reach men at highest risk and supports early detection of prostate cancer — enabling men to access PSA counselling and testing without burdening GPs. These services must be high quality, have appropriate clinical oversight, and communicate effectively with patients and primary/secondary care. Throughout, panellists modified statements mentioning ‘GPs’, replacing with ‘trained health professionals’ to reflect the broader workforce (for example, general practice nurses) involved in primary care service delivery. It was agreed that PSA counselling information can come from ‘trained health professionals’ and charities, or can be delivered through validated online tools to support informed choice. Current initiatives, commissioned by NHS England as pilots, to help tackle the impact of COVID-19 on referrals and men starting treatment for prostate cancer also aim to address inequalities of access to health care and PSA testing.^33^ These initiatives have demonstrated how proactive approaches can work in practice; however, outcomes from these pilots are yet to be formally reported.

Evidence suggests DRE has limited benefit as a screening tool in primary care,^21^^,^^22^ and is a known barrier for some men coming forwards for prostate health checks, believing it is the first and only test for prostate cancer or feel anxiety, shame, or embarrassment about it. Although DRE is still considered useful in men presenting with symptoms, for asymptomatic men, panellists believe that with MRI in the diagnostic pathway, DRE is not necessary and a PSA ≥3.0 ng/mL alone is sufficient to refer.

## Data

All data sets have been included either in the main article or supplementary information.
